# Prognostic value of emotional distress in advanced non–small cell lung cancer: a systematic review and meta-analysis

**DOI:** 10.3389/fonc.2026.1836242

**Published:** 2026-05-20

**Authors:** Jia-jia Lin, Rui Zhou, Zi-yan He, Tian-lin Wang, Ze-xin Zhang, Yan-juan Zhu, Yi-han He, Xue-song Chang, Ya-dong Chen, Hai-bo Zhang

**Affiliations:** 1 The Second Clinical College of Guangzhou University of Chinese Medicine, Guangzhou, China; 2 Department of Oncology, The Second Affiliated Hospital of Guangzhou University of Chinese Medicine, Guangdong Provincial Hospital of Traditional Chinese Medicine, Guangzhou, China; 3 Guangdong-Hong Kong-Macau Joint Lab on Chinese Medicine and Immune Disease Research, Guangzhou University of Chinese Medicine, Guangzhou, China; 4 Guangdong Provincial Key Laboratory of Clinical Research on Traditional Chinese Medicine Syndrome, Guangzhou, China; 5 Guangzhou Key Laboratory of Acupuncture and Medicine Research, Guangzhou, China

**Keywords:** emotional distress, immune checkpoint inhibitors, meta-analysis, non-small cell lung cancer, prognosis

## Abstract

**Objective:**

To evaluate the association between emotional distress (ED) and the efficacy of anti-tumor therapies in advanced non-small cell lung cancer (NSCLC).

**Methods:**

Eligible studies were cohort studies investigating the association between ED and treatment outcomes in patients with advanced NSCLC receiving anti-tumor therapies. All statistical analyses were performed using RevMan 5.4, R 4.2.3, and Stata 13.0.

**Results:**

Eight studies involving 911 patients were included, and the overall risk of bias was judged to be acceptable. Two studies involved treatment regimens based on immune checkpoint inhibitors (ICIs), another two focused solely on chemotherapy, while the remaining four included patients receiving various therapies involving chemotherapy, radiotherapy, or targeted therapy. Meta-analysis showed that ED was significantly associated with reduced overall survival (OS) (HR = 1.85, 95% CI: 1.50–2.28), an increased risk of progression (HR = 1.80, 95% CI: 1.22–2.66), and a lower objective response rate (ORR) (OR = 0.55, 95% CI: 0.37–0.80). These associations were generally consistent across treatment subgroups, without significant interaction effects. Sensitivity analysis supported the stability of the OS results. Additionally, subgroup analyses did not demonstrate statistically significant differences by age or timing of ED assessment, although the direction of effects was consistent. The certainty of evidence was low for OS and ORR, and very low for PFS.

**Conclusions:**

ED is significantly associated with poorer prognosis in patients with advanced NSCLC undergoing active anti-tumor therapies, contributing to both reduced short-term efficacy and diminished long-term survival. Further research should focus on elucidating the underlying mechanisms and exploring effective strategies to improve clinical outcomes in patients with advanced NSCLC and ED.

## 1 Introduction

The 5-year survival rate for advanced non-small cell lung cancer (NSCLC) remains below 20%, making it a leading cause of cancer-related mortality worldwide (1, 2). Current first-line treatment strategies for advanced NSCLC encompass chemotherapy, targeted therapy, immunotherapy, and local radiotherapy. The advent of targeted therapy and immune checkpoint inhibitors (ICIs) has significantly improved treatment response rates and survival outcomes (3). Nevertheless, heterogeneity in treatment response and the emergence of acquired resistance continue to pose major clinical challenges (2). Identifying factors that modulate the effectiveness of modern therapies is therefore of substantial importance.

In recent years, psychological factors, in addition to tumor genomics, have increasingly been recognized as potential modulators of cancer outcomes. Emotional distress (ED) refers to a persistent negative emotional state experienced by individuals facing multiple stressors, often manifesting as multidimensional psychological responses including depression, anxiety, and chronic stress (4, 5). Multiple studies indicate that the prevalence of ED among patients with lung cancer ranges from 33% to 51% (6, 7). Moreover, ED has been widely linked to adverse outcomes across various malignancies, including breast cancer (8) and glioma (9). However, its prognostic significance in advanced NSCLC remains inconclusive, with two recent large-scale studies reporting conflicting results (10, 11). These inconsistencies may reflect heterogeneity in patient selection, as some studies may have included individuals who did not receive systemic therapy, making it difficult to distinguish the independent effects of ED on treatment decision-making from those on treatment efficacy. To date, no systematic review has specifically evaluated the association between ED and treatment outcomes among patients with advanced NSCLC receiving anti-tumor therapies.

Notably, most previous studies investigating the prognostic impact of ED in oncology have primarily focused on patients undergoing conventional chemotherapy and radiotherapy. Advanced NSCLC has now clearly entered the era of precision medicine, driven by targeted therapies and ICIs, resulting in profound changes in the mechanisms of treatment response. Preclinical evidence suggests that stress hormones associated with ED may impair anti-tumor immunity and promote resistance to targeted agents (12–14), prompting interest in whether the impact of ED differs according to treatment modality in the current era of precision medicine. However, no meta-analysis to date has evaluated this association.

Accordingly, we conducted a systematic review and meta-analysis to examine the association between ED and the risks of disease progression and mortality among patients with advanced NSCLC receiving anti-tumor therapy, and to evaluate its prognostic significance across different treatment modalities. Our study aimed to elucidate the complex influence of ED on treatment outcomes, thereby providing valuable insights for clinical management and future research in psycho-oncology.

## 2 Methods

### 2.1 Search strategy

The protocol for this meta-analysis was registered in PROSPERO (CRD420251017845) and conducted in accordance with the Preferred Reporting Items for Systematic Reviews and Meta-Analyses (PRISMA) 2020 guidelines and flow diagram (15). Five databases (PubMed, Embase, the Cochrane Library, Web of Science and PsycINFO) were systematically searched from inception to April 18, 2026. The search strategy consisted of 3 key terms: NSCLC, ED (including depression, anxiety, stress, mental disorder, and psychological distress), and clinical outcome (including survival, mortality, prognosis). Detailed search terms for each database are provided in **Supplementary Table 1**-**5**. In addition, reference lists of relevant articles were manually screened, through which one eligible study that was indexed in the databases but not captured by the predefined search strategy was identified and included.

### 2.2 Inclusion and exclusion criteria

Two investigators (JJL and RZ) independently screened the records of comprehensive searches based on the following inclusion criteria: 1) Trials with a study population of patients who had histologically or cytologically confirmed locally advanced or metastatic NSCLC; 2) Trials with ED assessment conducted by standardized psychometric questionnaires or structured clinical interviews; 3) Trials with patients receiving anti-tumor treatment; 4)Trials with retrospective or prospective evaluation of the association between ED and survival outcomes; 5) Trials with efficacy outcomes of overall survival (OS), progression-free survival (PFS), or objective response rate (ORR).

The exclusion criteria were as follows: 1) Studies involving patients with severe psychiatric disorders (e.g., schizophrenia); 2) Studies not published in English; 3) Duplicate studies. Additionally, in cases where multiple publications originated from the same or overlapping patient cohorts, only the most recent and/or most comprehensive study was included in the meta-analysis.

### 2.3 Data extraction and collection

Two investigators (JJL and RZ) independently extracted data using a predefined form. The extracted information included: first author’s name, publication year, country, beginning year of the trial, publication type, mean age, number of participants, gender distribution, NSCLC stage, treatment details, ED type, ED definition, ED assessment timing, and major confounders adjusted for. Study authors were contacted when key data were missing or unclear.

### 2.4 Quality assessment

Methodological quality was assessed using validated tools tailored to the specific study designs. For observational studies, quality was assessed using the Newcastle–Ottawa Scale (NOS), which evaluates the domains of selection, comparability, and outcome. Each study received a methodological score from 0 (lowest) to 9 (highest) according to the NOS, and scores were classified as high (≥7 stars), moderate (4–6 stars), or low (<4 stars). For randomized controlled trials (RCTs), the Cochrane Risk of Bias tool version 2 (RoB 2) was used to evaluate methodological quality. Each RCT was judged as having a low, some concerns, or high risk of bias across five domains: bias arising from the randomization process, deviations from intended interventions, missing outcome data, measurement of the outcome, and selection of the reported result. Two investigators (ZYH and TLW) independently assessed study quality, and any discrepancies were resolved through discussion with a third author (HBZ).

### 2.5 Statistical analysis

All statistical analyses were performed using RevMan (version 5.4; The Cochrane Collaboration), R (version 4.2.3; R Foundation for Statistical Computing, Vienna, Austria). and Stata (version 13.0; StataCorp, College Station, TX, USA). A two-tailed P-value < 0.05 was considered statistically significant. Associations between ED and clinical outcomes were quantified using pooled effect estimates. Specifically, hazard ratios (HRs) with 95% confidence intervals (CIs) were calculated for OS and PFS, while odds ratios (ORs) with 95% CIs were computed to evaluate differences in ORR. An established algebraic method was applied to estimate the lnHR and its standard error (selnHR) from the reported event counts and log-rank p-values when direct estimates were unavailable (16, 17).

Heterogeneity was evaluated using the I^2^ statistic and Cochran’s Q test. A random-effects model was applied when heterogeneity was substantial (I² ≥ 50% or P < 0.10); otherwise, a fixed-effects model was applied. Subgroup analyses were carried out to investigate the association of ED with efficacy outcomes (OS, PFS, ORR) stratified by treatment regimen. Additionally, subgroup analysis of OS by the timing of ED assessment and age was performed to explore potential effect modification. Publication bias was planned to be assessed using funnel plots if at least 10 studies were included in any individual meta-analysis. Sensitivity analyses were conducted by sequentially removing individual studies to evaluate the robustness of the pooled estimates.

### 2.6 Certainty of evidence assessment

The certainty of evidence for the three major outcomes (OS, PFS, and ORR) was independently evaluated by two investigators (ZYH and TLW) using the Grading of Recommendations, Assessment, Development, and Evaluation (GRADE) approach. Following GRADE guidance, the evidence was assessed across five domains: risk of bias, inconsistency, indirectness, imprecision, and publication bias. The certainty of evidence for each outcome was categorized as high, moderate, low, or very low. Any discrepancies were resolved through discussion or consultation with a third author (HBZ).

## 3 Results

### 3.1 Study selection and characteristics

**Figure 1:**
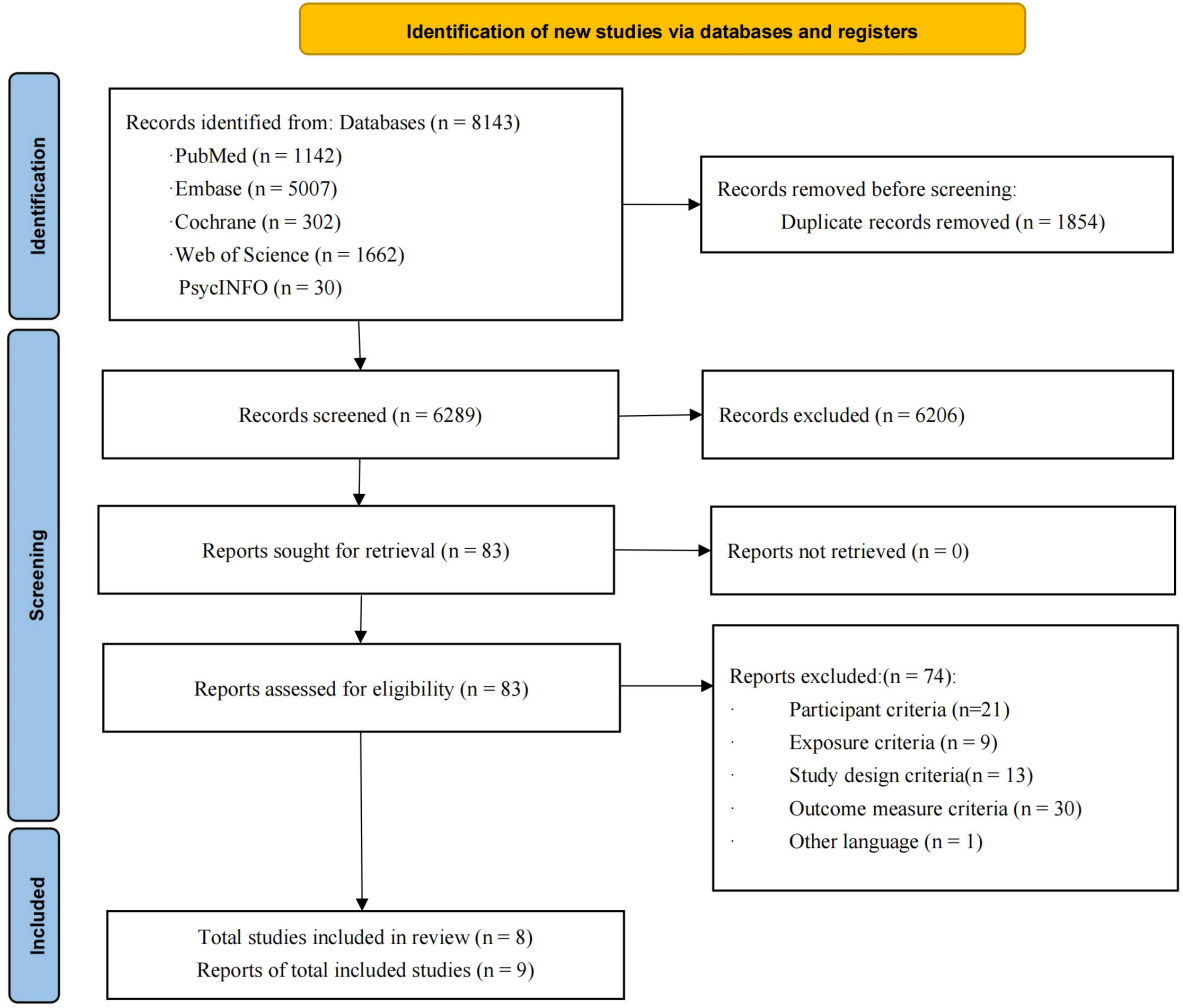
Flowchart of study selection.

A total of 8,143 records were identified through database searches. After removing 1,854 duplicates, 6,289 records remained for title and abstract screening. Of these, 83 articles were assessed for full-text eligibility. Finally, 9 reports from 8 studies meeting predefined inclusion criteria were retained for final analysis, involving a total of 911 patients (18–26) (**Figure 1**).

The characteristics of the included studies are summarized in **Table 1**. The largest number of studies were conducted in China (n = 4) and the United States (n = 2). Two studies involved treatment regimens based on ICIs, another two focused solely on chemotherapy, while the remaining four included patients receiving various therapies involving chemotherapy, radiotherapy, or targeted therapy. Among the included studies, depression was the most commonly evaluated type of ED (n = 6). ED was assessed at baseline in seven studies, including one study that also assessed ED during treatment, and one additional study assessed ED only during treatment. Regarding age, six studies included patients with a mean or median age over 60 years, whereas two included those with a mean/median age below 60 years. In addition, most studies adjusted for potential confounders that may affect outcome associations, principally including age, sex, tumor stage, and performance status.

**Table 1:** Characteristics of clinical studies included in the analysis.

First author (pub year), country	Beginning year	Publication type	Number of participants (ED/no ED)	Gender (M/F)	Mean age	Staging	Treatment	ED type	ED definition	ED assessment timing^1^	Major confounders adjusted for	Outcome
Tatsuo Akechi (2001), Japan (25)	1996	Prospective cohort study	17/72	64/25	62	III-IV	Chemotherapy/radiation	Psychiatric Disorders	SCID	Baseline	/	ORR
William F. Pirl (2008), the US (21)	2003	Prospective cohort study	10/33	17/26	65.6	IIIB-IV	Chemotherapy/radiation/EGFR-TKI	Depression	HADSes	Baseline	Performance status	OS
Mei-Ling Chen (2011), China (23)	2005	Prospective cohort study	20/70	56/34	58.67 (median)	III-IV	Chemotherapy/Targeted therapy	Depression	HADSss	During treatment^2^	Age, gender, smoking status, disease stage, dyspnea symptom, and functional performance status	OS
William F. Pirl(2012), the US (22)	2006	RCT	21/129	72/78	64.4	IV	Chemotherapy ± radiation/EGFR-TKI	Major depression syndrome	PHQ-9 following DSM-IV guidelines	Baseline	Performance status, age, sex, race, marital status, and smoking history	OS
Oscar Arrieta (2013), Mexico (19)	2008	Retro- spective cohort study	27/55	48/34	58.9	IIIB-IV	Chemotherapy	Depression	HADSss; MINI	Baseline	Age, gender, smoking history, hypertension, histology, clinical stage, and Karnofsky performance status	OS
Jue Chen (2015), China (20)	2010	Prospective cohort study	48/78	73/53	60.7	IIIB-IV	Chemotherapy	Depression	Z-SDSsio	Baseline	/	OS/PFS/ORR
Zi-Ran Bi(2022), China (24)	2019	Prospective cohort study	50/54	84/20	64.44/ 66.50	III-IV	ICIs± chemotherapy	Psychologi-cal stress	DTre	Baseline	/	PFS /ORR
Yue Zeng (2024), China (18)	2021	Prospective cohort study	111/116	210/17	63 (median)	IIIB-IV	ICIs± chemotherapy	Depression and/or anxiety	PHQ-9ty and/or GAD-7ry	Baseline and during treatment^3^	Age, sex, histology, disease stage, PD-L1 expression, ECOG PS, smoking status, treatment regimen, brain/liver metastases, BMI, NLR	OS^4^/PFS/ORR

The US, the United States; RCT, Randomized Controlled Trial;/, not stated; EGFR-TKI, Epidermal Growth Factor Receptor Tyrosine Kinase Inhibitor; ICIs, Immune Checkpoint Inhibitors; SCID, Structured Clinical Interview for Diagnostic and Statistical Manual of Mental Disorders (3rd edition criteria); HADS, Hospital Anxiety and Depression Scale; DSM-IV, Diagnostic and Statistical Manual of Mental Disorders (4th edition criteria); MINI, Mini-International Neuropsychiatric Interview; ICD-9, International Classification of Diseases (9th revision); EMR, Electronic Medical Records; Z-SDS, Zung Self-Rating Depression Scale; DT, Distress Thermometer; PHQ-9, Patient Health Questionnaire-9; GAD-7, Generalized Anxiety Disorder 7-item; CCI, Charlson Comorbidity Index; PD-L1, Programmed Death Ligand 1; ECOG PS, Eastern Cooperative Oncology Group Performance Status; BMI, Body Mass Index; NLR, Neutrophil-to-Lymphocyte Ratio; OS, Overall Survival; ORR, Objective Response Rate; PFS, Progression-Free Survival.

^1,^ ED assessment timing referred to the time point(s) at which ED was measured for survival or other efficacy analyses.

^2^ 2 weeks after first-line treatment.

^3^ After 2 cycles of ICIs.

^4^ For the association between baseline ED and OS, updated results from a 2025 conference abstract of the same cohort were used.

**Supplementary Tables 6**, **7** and **Supplementary Figures 1**, **2** present the detailed and visualized risk of bias assessments. The seven cohort studies were evaluated using the NOS, with four rated as high quality and three as moderate quality. The randomized controlled trial was assessed using RoB 2 tool and was judged to have some concerns.

### 3.2 Association between ED and efficacy in advanced NSCLC

#### 3.2.1 Overall survival

**Figure 2:**
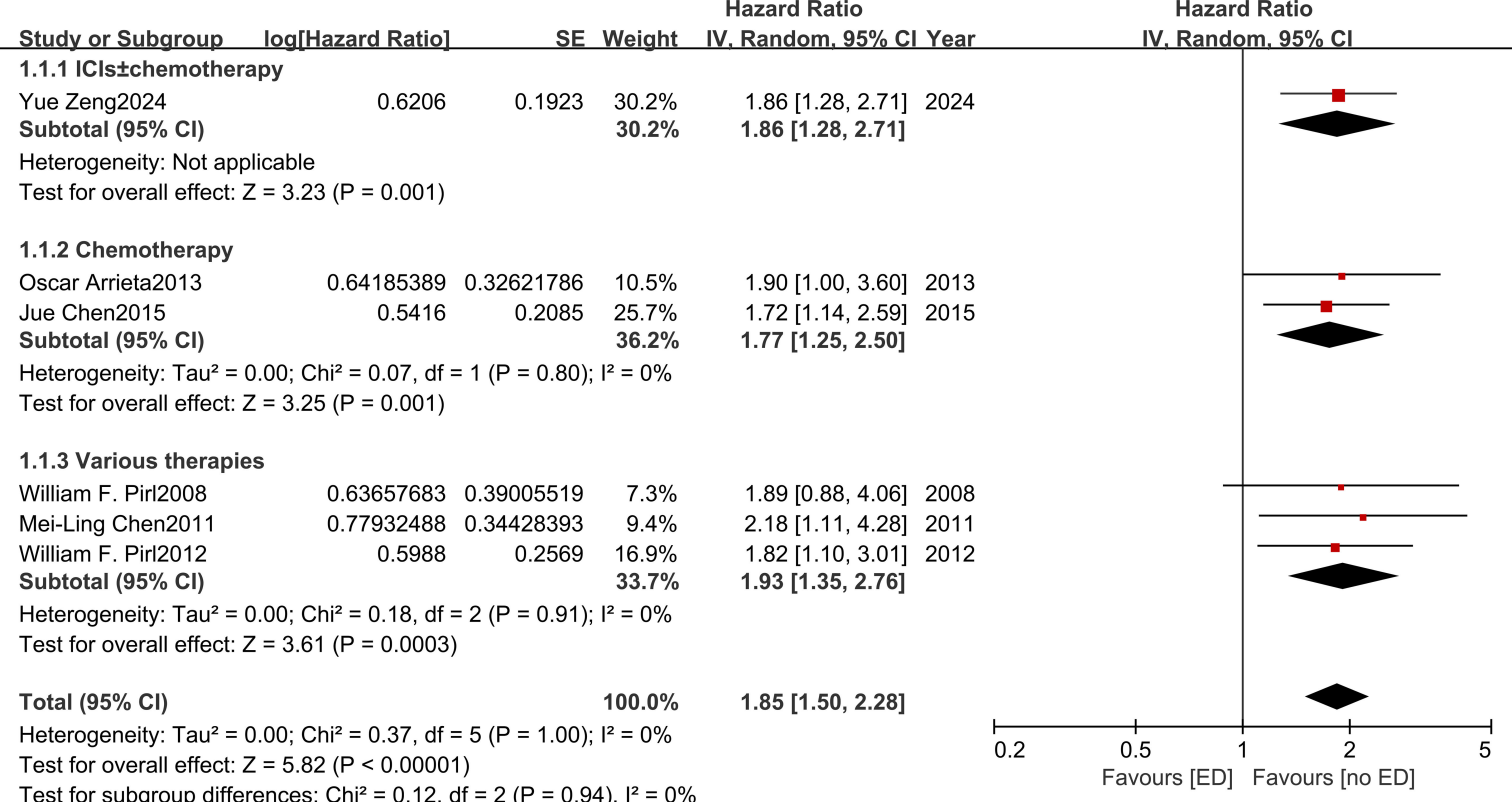
The effects of emotional distress on overall survival in patients with advanced non-small cell lung cancer.

Six studies (18–23, 26) reported overall survival OS and yielded a pooled HR of 1.85 (95% CI: 1.50–2.28; *I^2^* = 0%) (**Figure 2**) for the association between ED and OS based on fixed-effects model analysis. In subgroup analyses by treatment regimen, one study evaluated an ICI-based regimen (ICI ± chemotherapy) (HR 1.86, 95% CI 1.28–2.71; P = 0.001), two studies involved chemotherapy alone (HR 1.77, 95% CI 1.25–2.50; *I^2^* = 0%), and the remaining studies included various therapies (chemotherapy, radiotherapy, or targeted therapy) according to clinical conditions (HR 1.93, 95% CI 1.35–2.76; *I^2^* = 0%). No significant interaction was observed across treatment regimens (χ² = 0.12, df = 2, P = 0.94; *I^2^* = 0%).

Subgroup analyses by timing of ED assessment showed that ED was associated with shorter OS irrespective of assessment timing, with consistent effects observed at baseline (n = 5; HR 1.82, 95% CI 1.46–2.26; *I^2^* = 0%) and during treatment (n = 2; HR 2.12, 95% CI 1.38–3.27; *I^2^* = 0%). No significant between-subgroup difference was observed (χ² = 0.40, df = 1, P = 0.53; *I^2^* = 0%) (**Supplementary Figure 3**). Age-stratified analyses showed consistent associations, with pooled HRs of 1.81 (95% CI 1.43–2.28; *I^2^* = 0%) in patients aged >60 years (n = 4) and 2.03 (95% CI 1.27–3.23; *I^2^* = 0%) in those aged ≤60 years (n = 2), with no significant interaction by age (χ² = 0.19, df = 1, P = 0.66; *I^2^* = 0%) (**Supplementary Figure 4**).

#### 3.2.2 Progression-free survival

**Figure 3:**
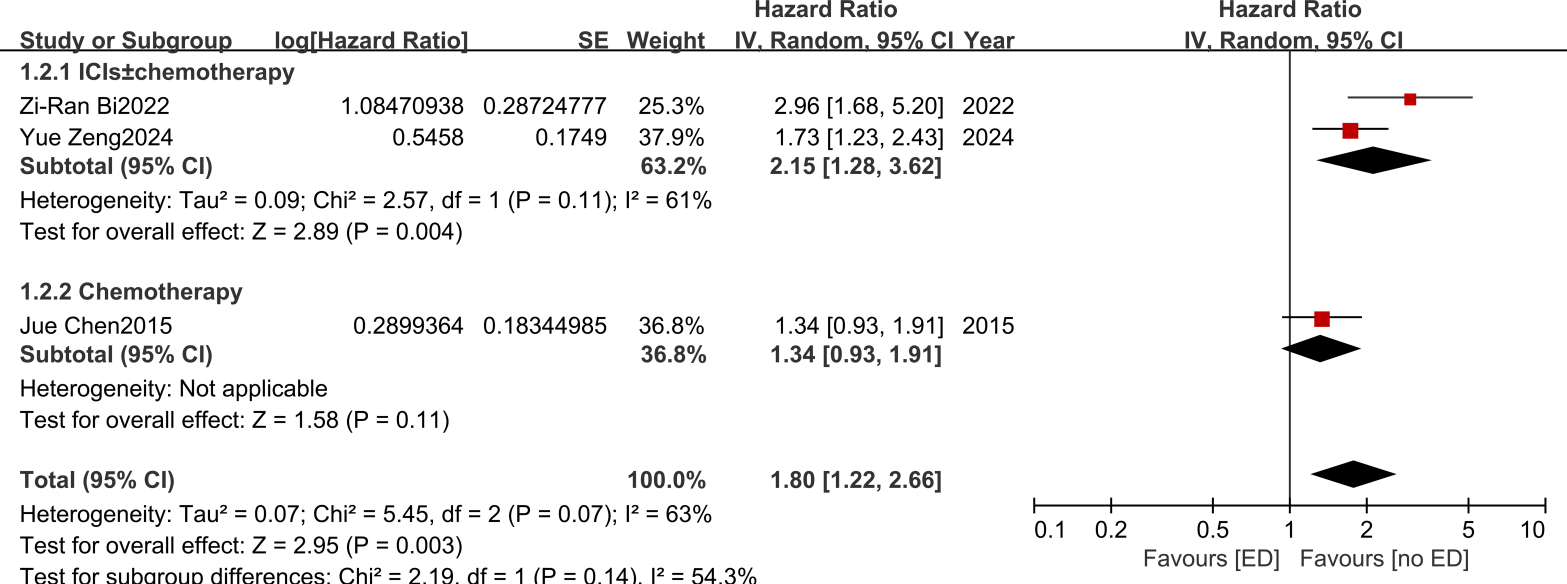
The effects of emotional distress on progression-free survival in patients with advanced non-small cell lung cancer.

Three studies (18, 20, 24) were included in the meta-analysis of PFS. Using a random-effects model, the pooled analysis yielded a HR of 1.80 (95% CI: 1.22–2.66; *I²* = 63%) (**Figure 3**). Subgroup analyses based on treatment regimens demonstrated that patients receiving ICIs ± chemotherapy had a higher risk of disease progression (n = 2; HR = 2.15, 95% CI: 1.28–3.62). In contrast, the association did not reach statistical significance in patients treated with chemotherapy alone (n = 1; HR = 1.34, 95% CI: 0.93–1.91). The test for subgroup differences did not indicate a statistically significant interaction (χ² = 2.19, df = 1, P = 0.14, *I^2^* = 54.3%).

#### 3.2.3 Objective response rate

**Figure 4:**
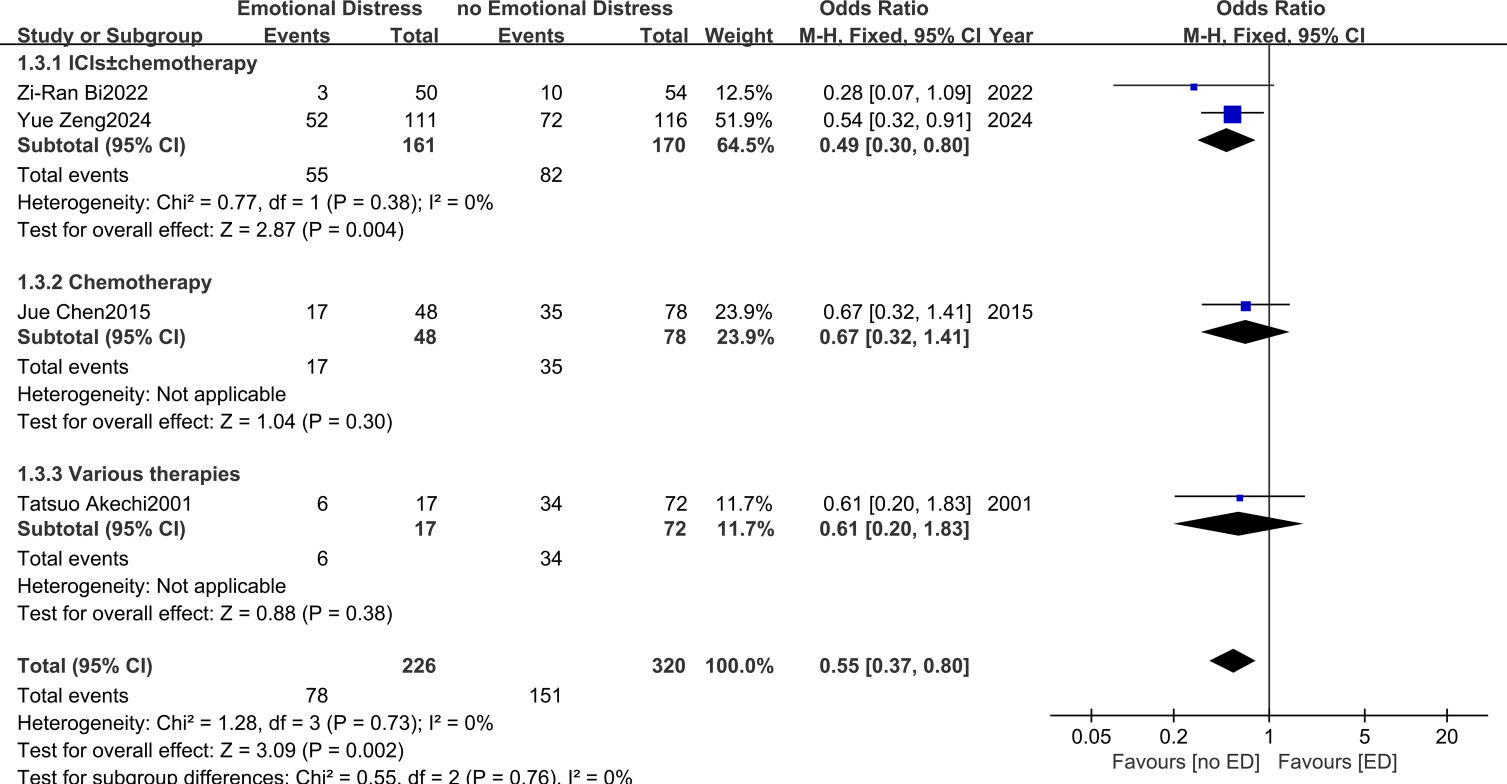
The effects of emotional distress on objective response rate in patients with advanced non-small cell lung cancer.

Data for the ORR meta-analysis were derived from four studies (18, 20, 24, 25). The fixed-effects meta-analysis revealed that patients with ED were significantly less likely to achieve an objective response than those without ED (OR = 0.55, 95% CI: 0.37–0.80; *I^2^* = 0%) (**Figure 4**). Subgroup analysis showed that ED was significantly associated with lower ORR among patients receiving ICIs ± chemotherapy (n = 2; OR = 0.54, 95% CI: 0.32–0.91; *I^2^* = 0%). Similar trends were observed in the chemotherapy alone group (n = 1; OR = 0.67, 95% CI: 0.32–1.41) and the group receiving various treatments (n = 1; OR = 0.61, 95% CI: 0.20–1.83), although these results did not reach statistical significance. The test for subgroup differences indicated no statistically significant heterogeneity across subgroups (χ² = 0.55, df = 2, P = 0.76; *I^2^* = 0%). Overall, ED was significantly associated with a reduced ORR in patients with advanced NSCLC.

### 3.3 Publication bias and sensitivity analysis

Due to the limited number of studies (<10) in each analysis, evaluation of publication bias was not performed. Sensitivity analyses for OS were conducted to assess the stability of the results by sequentially removing one study at a time. The results showed that the corresponding pooled estimates were not substantially changed, indicating that the findings were not unduly influenced by any individual study (**Supplementary Figure 5**).

### 3.4 Certainty of evidence

The certainty of evidence for all reported outcomes was evaluated using the GRADE framework (**Supplementary Table 7**). Because the included studies were mainly observational cohort studies, all outcomes started at low certainty. The certainty of evidence remained low for OS and ORR, whereas that for PFS was downgraded to very low because of serious risk of bias and substantial heterogeneity.

## 4 Discussion

This meta-analysis elucidates the association between ED and treatment efficacy in patients with advanced NSCLC. Some prior studies suggest that ED may indirectly affect clinical outcomes in patients with advanced NSCLC by reducing treatment uptake (10, 27). We intentionally focused on patients who had received active anti-cancer therapy thereby aiming to isolate the prognostic impact of ED from survival disadvantages introduced by impaired treatment decision-making and access. Our findings further demonstrate that ED is independently associated with shorter overall survival and progression-free survival in treated patients with advanced NSCLC, indicating that ED is more likely to adversely affect survival by directly compromising the efficacy of delivered anticancer treatments.

Most of the primary studies included in our meta-analysis adjusted for lifestyle factors (e.g., smoking) and disease-related variables (e.g., tumor stage and performance status), thereby enhancing the internal validity of the observed association between ED and reduced treatment efficacy. Nevertheless, in real-world clinical practice, the onset of ED and its association with adverse outcomes are likely driven by multiple interacting factors. In terms of lifestyle, patients experiencing ED are more susceptible to engaging in unhealthy behaviors such as smoking and hazardous alcohol consumption (8). These habits may further compromise immune function and ultimately elevate the risk of tumor progression and mortality (28). Tobacco use is established as the predominant etiological factor for lung cancer (29), and current evidence in cancer populations indicates that current smoking is independently associated with higher levels of depression and overall symptom burden (30, 31). In parallel, studies have shown that higher anxiety levels are associated with a greater proportion of high-risk drinking behaviors among cancer patients, such patterns of alcohol may adversely affect treatment tolerance and adherence (32). Regarding disease-related factors, patients with poorer baseline performance status and higher tumor burden tend to exhibit more pronounced depressive and anxiety symptoms (33); notably, these factors independently predict inferior survival outcomes. Furthermore, clinical studies have found that the incidence of anxiety and depression significantly increases after brain radiotherapy (34). Radiation-induced neuroinflammation, blood-brain barrier disruption, and injury to frontal-subcortical circuits are considered key pathophysiological mechanisms leading to these psychiatric symptoms (34). Similarly, prolonged use of certain supportive care medications commonly prescribed in oncology practice, such as glucocorticoids, has been associated with an increased risk of depressive and anxiety symptoms (35). Taken together, these multidimensional pathways suggest a bidirectional relationship: the malignancy and its requisite treatments may aggravate emotional distress, while ED may reciprocally attenuate therapeutic efficacy through intertwined behavioral and biological mechanisms.

To further elucidate the interaction between ED and targeted or immunotherapy, we performed a subgroup analysis based on treatment regimens. Among patients receiving ICI-based therapies, ED was associated with poorer OS, PFS, and ORR. This observation may be partially explained by the reliance of ICI efficacy on an intact anti-tumor immune response, which may be compromised in the presence of ED (18). Preclinical studies have shown that ED persistently activates the HPA axis and the sympathetic nervous system, resulting in sustained elevations of endogenous glucocorticoids and catecholamines, which subsequently suppress T lymphocyte activation and antigen presentation (36, 37). Meanwhile, chronic stress increases the infiltration of immunosuppressive cells such as myeloid-derived suppressor cells (MDSCs) and tumor-associated macrophages (TAMs), while simultaneously dampening the effector functions of CD8^+^ T cells and natural killer (NK) cells (38). Acute stress may induce T cell dysfunction and exhaustion through aberrant activation of the kisspeptin/GPR54 signaling pathway. Moreover, sustained elevation of pro-inflammatory cytokines such as interleukin-6 (IL-6) in ED may disrupt immune homeostasis and has been associated with reduced responsiveness to PD-1 inhibitors (39, 40). These multi-level mechanisms collectively drive an immunosuppressive TME, ultimately reducing the clinical benefit of ICIs in patients with ED. Importantly, emerging evidence suggests that antidepressants may enhance CD8+ T cell effector function by inhibiting the serotonin transporter (SERT), thereby synergizing with PD-1 inhibitors to improve anti-tumor immunity (41, 42). Therefore, elucidating the regulatory network by which ED compromises ICI efficacy may provide potential therapeutic targets not only for improving immunotherapy responses but also for optimizing treatment strategies in patients with ED.

In the subgroup receiving various therapies, including targeted therapy, the pooled OS HR was 1.93 (95% CI: 1.35–2.76). While not exclusive to targeted therapy, this finding raises the possibility that ED may negatively influence treatment outcomes in this population. A recent multicenter retrospective study presented by Yang Z et al. at the World Conference on Lung Cancer investigated the impact of ED on the clinical efficacy of EGFR tyrosine kinase inhibitors (EGFR-TKIs) in patients with advanced NSCLC. The results showed that patients with coexisting ED had a significantly shorter median PFS (HR 1.70, 95% CI 1.20–2.51; P = 0.003), as well as a significantly lower ORR (OR 0.37, P = 0.031). Due to the limited completeness of data available in conference abstracts, this study was not included in the quantitative synthesis of the present meta-analysis; however, its findings further support a potential negative impact of ED on the clinical benefit of EGFR-TKI therapy. Mechanistically, Professor Monique B. Nilsson and colleagues at Memorial Sloan Kettering Cancer Center reported that chronic stress hormones can activate β2-ARs on tumor cells. These receptors interact with mutant EGFR signaling pathways, resulting in the inactivation of LKB1, the activation of CREB, and the overexpression of interleukin-6 (IL-6), which together contribute to the development of resistance to EGFR tyrosine kinase inhibitors (EGFR-TKIs) (14). Real-world evidence further revealed that EGFR-mutant patients receiving β-blockers exhibited lower serum IL-6 levels and prolonged PFS, underscoring the clinical modifiability of this pathway (14). Additionally, Jie Xie et al. reported that norepinephrine (NE) upregulates connexin 32 (Cx32) via CREB activation, elevating resistance-associated proteins (MET/IGF-1R) and promoting afatinib resistance (43). Given the increasingly prominent role of targeted therapy in the management of advanced NSCLC, further high-quality preclinical studies and prospective clinical evidence is warranted to clarify the specific influence of ED on targeted therapy efficacy.

Although the differences did not reach statistical significance, our results indicated that the associations between ED and OS may vary according to age and the timing of ED assessment. Specifically, a trend toward a higher mortality risk was observed in younger patients with ED compared with older patients with ED. Among patients aged ≤60 years with advanced NSCLC, ED was associated with a 103% increase in mortality risk relative to those without ED. This may be related to heavier family burdens and more complex social roles among younger cancer patients (44). Furthermore, current evidence shows that younger patients often report higher prevalence and severity of ED, which may reduce treatment efficacy through mechanisms such as heightened stress responses or accelerated immune dysfunction (45). In addition, younger patients differ from older patients in terms of metabolic status, immune function, and treatment responses. These differences may have a synergistic effect, amplifying the adverse impact of ED on survival outcomes in younger patients with NSCLC (46).

Regarding the timing of ED assessment, our meta-analysis suggested that ED assessed during treatment may show a stronger trend toward an association with poorer OS compared with ED assessed at baseline. This pattern may be partially explained by the fact that baseline ED is often influenced by acute psychological responses to cancer diagnosis, whereas ED assessed during treatment may better reflect a more persistent and stable psychological state (47). Existing longitudinal studies have demonstrated that psychological distress in patients with lung cancer fluctuates over the disease course (48). Moreover, a bidirectional relationship between ED and treatment outcomes is plausible, whereby poor treatment response or treatment-related adverse events may further contribute to the onset or exacerbation of (49). Taken together, future prospective studies should incorporate repeated, longitudinal assessments of ED to better characterize its dynamic trajectory and to explore the most clinically informative assessment window.

### 4.1 Clinical implications and future direction

Collectively, our findings indicate that ED is associated with adverse clinical outcomes in patients with advanced NSCLC undergoing active anti-tumor treatment. Although exploratory subgroup analyses suggested a potentially stronger association between ED and treatment outcomes among patients receiving ICI-based regimens, no statistically significant interaction across treatment modalities was observed. Overall, ED should be considered a clinically relevant factor associated with treatment outcomes.

From a clinical practice perspective, these findings support the rationale for implementing routine screening and longitudinal assessment of ED in patients with advanced NSCLC. The National Comprehensive Cancer Network (NCCN) recommends integrating ED assessment into standard oncological care as the “sixth vital sign” (50, 51). With this framework, the NCCN Distress Thermometer (DT) is widely utilized as an initial screening tool. Patients who screen positive (DT score ≥4) should undergo further evaluation using validated instruments, such as the PHQ-9, GAD-7, or HADS, tailored to the predominant symptom profile (51). Furthermore, a stepped-care model based on symptom severity is further recommended (51). Patients with mild distress may be managed with supportive care delivered by the oncology team, whereas those with moderate-to-severe distress warrant prompt referral to specialized psycho-oncology or psychiatric services (50).

For patients with clinically significant ED, evidence-based interventions may be considered. High-level evidence from RCTs and meta-analyses indicates that cognitive behavioral therapy (CBT) and mindfulness-based cognitive therapy (MBCT) can significantly alleviate ED and improve quality of life (52, 53). Regarding pharmacological approaches, recent evidence suggests that selective serotonin reuptake inhibitors (SSRIs) may not only ameliorate depressive symptoms but also potentially enhance T-cell-mediated antitumor immunity via SERT regulation, providing a potential biological rationale for interaction with ICIs (41). Importantly, the present meta-analysis did not evaluate the effects of psychological or pharmacological intervention. Moreover, high-quality randomized evidence linking ED-targeted interventions to improved anti-tumor efficacy remains limited. Future well-designed, adequately powered RCTs are warranted to address this gap.

### 4.2 Study limitations

Several limitations of this meta-analysis should be acknowledged. First, no clinical studies specifically investigating the association between ED and the efficacy of targeted therapy in advanced NSCLC were eligible for inclusion in our meta-analysis. Most of the available evidence remains limited to preclinical studies, making it difficult to directly compare the potential impact of ED across different treatment modalities. Second, there was a large variation in the definitions of ED across studies. Additionally, the methods, sensitivity, scale structure, and cutoff values of the tools used to assess ED were inconsistent, so we were unable to conduct further subgroup analyses based on different ED types or compare dose–response associations between ED and treatment efficacy.

## 5 Conclusion

In this systematic review and meta-analysis, a significant association between ED and poorer prognosis was observed in NSCLC patients undergoing active anti-cancer therapies, with ED reducing short-term treatment efficacy and decreasing long-term survival. These associations were generally consistent across treatment subgroups. These findings suggest a potential need for early detection and management of ED in clinical practice and support its consideration as a patient-reported outcome (PRO) in trials evaluating new treatment regimens. Further high-quality studies are warranted to determine the most effective intervention strategies for improving treatment outcomes in this population and to elucidate the underlying mechanisms linking ED to poorer survival.

## Data Availability

The original contributions presented in the study are included in the article/**Supplementary Material**. Further inquiries can be directed to the corresponding authors.

## Supplementary material

Supplementary Table 1 Search strategy for Pubmed.

Supplementary Table 2 Search strategy for Embase.

Supplementary Table 3 Search strategy for Cohorane.

Supplementary Table 4 Search strategy for Web of Science.

Supplementary Table 5 Search strategy for psycINFO.

Supplementary Table 6 Quality assessment of studies included according to the Newcastle–Ottawa Scale.

Supplementary Table 7 Quality assessment of randomized controlled trials included according to the Cochrane Risk of Bias 2.0 tool.

Supplementary Table 8 Evidence profile for emotional distress and clinical outcomes in advanced non-small cell lung cancer.

Supplementary Figure 1 Quality assessment of studies included according to the Newcastle–Ottawa Scale.

Supplementary Figure 2 Quality assessment of randomized controlled trials included according to the Cochrane Risk of Bias 2.0 tool.

Supplementary Figure 3 Effect of emotional distress on overall survival by timing of ED assessment.

Supplementary Figure 4 Effect of emotional distress on overall survival by age subgroup.

Supplementary Figure 5 Sensitivity analysis for overall survival.
