# Supplementary material for: Prognostic value of emotional distress in advanced non–small cell lung cancer: a systematic review and meta-analysis
Source: Front Oncol. 2026 May 20;16:1836242. doi: 10.3389/fonc.2026.1836242 (PMC13230033; doi:10.3389/fonc.2026.1836242)
Supplement: Supplementary Table 1 — Search strategy for Pubmed. [file Table1.docx]

**A systematic review and meta-analysis on the prognostic value of emotional distress in non-small cell lung cancer patients**

| **Legend** |  | **Pages** |
| --- | --- | --- |
| Supplementary Table S1 | Search strategy for Pubmed | 2 |
| Supplementary Table S2 | Search strategy for Embase | 3 |
| Supplementary Table S3 | Search strategy for Cohorane | 4 |
| Supplementary Table S4 | Search strategy for Web of Science | 5 |
| Supplementary Table S5 | Search strategy for psycINFO | 6 |
| Supplementary Table S6 | Quality assessment of studies included according to the Newcastle–Ottawa Scale | 7 |
| Supplementary Table S7 | Quality assessment of randomized controlled trials included according to the Cochrane Risk of Bias 2.0 tool | 8 |
| Supplementary Table S8 | Evidence profile for emotional distress and clinical outcomes in advanced non-small cell lung cancer | 9 |

**Supplementary Table S1. Search strategy for Pubmed**

| #1 | ("Carcinoma, Non-Small-Cell Lung"[MeSH Terms] OR "Carcinoma, Non-Small-Cell Lung"[tiab] OR "Carcinoma, Non Small Cell Lung"[tiab] OR "Non-Small-Cell Lung Carcinomas"[tiab] OR "Carcinoma, Non-Small Cell Lung"[tiab] OR "Non-Small Cell Lung Cancer"[tiab] OR "Non-Small-Cell Lung Carcinoma"[tiab] OR "Non Small Cell Lung Carcinoma"[tiab] OR "Nonsmall Cell Lung Cancer"[tiab] OR "Non-Small Cell Lung Carcinoma"[tiab] OR "Non small Cell Lung Cancer"[tiab] OR "NSCLC"[tiab]) AND (("Survival"[MeSH Terms] OR "Mortality"[MeSH Terms] OR "Prognosis"[MeSH Terms] OR "Survival Analysis"[MeSH Terms] OR "Risk Assessment"[MeSH Terms] OR survival[tiab] OR mortal*[tiab] OR death[tiab] OR prognos*[tiab] OR "hazard ratio"[tiab] OR "HR"[tiab] OR "overall survival"[tiab] OR "progression-free survival"[tiab] OR "Overall Response Rate"[tiab] OR "ORR"[tiab] OR "Disease Control Rate"[tiab] OR "DCR"[tiab])) AND (("Depression"[MeSH Terms] OR "Anxiety"[MeSH Terms] OR "Stress, Psychological"[MeSH Terms] OR "Mental Disorders"[MeSH Terms] OR depress*[tiab] OR anxiety[tiab] OR "chronic stress"[tiab] OR stress[tiab] OR "psychological distress"[tiab] OR "emotional distress"[tiab] OR "mental disorder"[tiab] OR "depressive disorder"[tiab])) | 1142 |
| --- | --- | --- |

**Supplementary Table S2.** **Search strategy for Embase**

| #1 | 'carcinoma, non-small-cell lung':ab,ti OR 'carcinoma, non small cell lung':ab,ti OR 'non-small-cell lung carcinomas':ab,ti OR 'carcinoma, non-small cell lung':ab,ti OR 'non-small cell lung cancer':ab,ti OR 'non-small-cell lung carcinoma':ab,ti OR 'non small cell lung carcinoma':ab,ti OR 'nonsmal cell lung cancer':ab,ti OR 'non-small cell lung carcinoma':ab,ti OR 'non small cell lung cancer':ab,ti OR nsclc:ab,ti | 191488 |
| --- | --- | --- |
| #2 | survival:ab,ti OR mortal*:ab,ti OR death:ab,ti OR prognos*:ab,ti OR 'hazard ratio':ab,ti OR hr:ab,ti OR 'overall survival':ab,ti OR 'progression-free survival':ab,ti OR 'overall response rate':ab,ti OR orr:ab,ti OR 'disease control rate':ab,ti OR dcr:ab,ti | 5,778,860 |
| #3 | depress*:ab,ti OR anxiety:ab,ti OR 'chronic stress':ab,ti OR stress:ab,ti OR 'psychological distress':ab,ti OR 'emotional distress':ab,ti OR 'mental disorder':ab,ti OR 'depressive disorder':ab,ti | 2,508,077 |
| #4 | 'survival'/exp | 1,729,387 |
| #5 | 'mortality'/exp | 1,704,710 |
| #6 | 'prognosis'/exp | 1,060,257 |
| #7 | 'survival analysis'/exp | 82,581 |
| #8 | 'risk assessment'/exp | 905,155 |
| #9 | 'depression'/exp | 829,374 |
| #10 | 'anxiety'/exp | 413,421 |
| #11 | 'mental stress'/exp | 270,553 |
| #12 | 'mental disease'/exp | 3,481,047 |
| #13 | 'non small cell lung cancer'/exp | 275,986 |
| #14 | #1 OR #13 | 294,473 |
| #15 | #2 OR #4 OR #5 OR #6 OR #7 OR #8 | 7,184,595 |
| #16 | #3 OR #9 OR #10 OR #11 OR #12 | 5,177,188 |
| #17 | #14 AND #15 AND #16 | 5,007 |

**Supplementary Table S3. Search strategy for Cochrane**

| #1 | MeSH descriptor: [Carcinoma, Non-Small-Cell Lung] explode all trees | 7039 |
| --- | --- | --- |
| #2 | MeSH descriptor: [Mortality] explode all trees | 18712 |
| #3 | MeSH descriptor: [Prognosis] explode all trees | 235809 |
| #4 | MeSH descriptor: [Survival] explode all trees | 176 |
| #5 | MeSH descriptor: [Survival Analysis] explode all trees | 29900 |
| #6 | MeSH descriptor: [Risk Assessment] explode all trees | 13881 |
| #7 | MeSH descriptor: [Depression] explode all trees | 20305 |
| #8 | MeSH descriptor: [Anxiety] explode all trees | 14307 |
| #9 | MeSH descriptor: [Stress, Psychological] explode all trees | 10173 |
| #10 | MeSH descriptor: [Mental Disorders] explode all trees | 115671 |
| #11 | MeSH descriptor: [Psychological Distress] explode all trees | 741 |
| #12 | (Carcinoma, Non-Small-Cell Lung):ti,ab,kw OR (Carcinoma, Non Small Cell Lung):ti,ab,kw OR (Non-Small-Cell Lung Carcinomas):ti,ab,kw OR (Carcinoma, Non-Small Cell Lung):ti,ab,kw OR (Non-Small Cell Lung Cancer):ti,ab,kw (Word variations have been searched) | 18695 |
| #13 | (Non-Small-Cell Lung Carcinoma):ti,ab,kw OR (Non Small Cell Lung Carcinoma):ti,ab,kw OR (Nonsmall Cell Lung Cancer):ti,ab,kw OR (Non-Small Cell Lung Carcinoma):ti,ab,kw OR (Non small Cell Lung Cancer):ti,ab,kw (Word variations have been searched) | 18980 |
| #14 | (NSCLC):ti,ab,kw (Word variations have been searched) | 13518 |
| #15 | #1 or #12 or #13 or #14 | 19861 |
| #16 | (survival):ti,ab,kw OR (mortal*):ti,ab,kw OR (death):ti,ab,kw OR (prognos*):ti,ab,kw OR (hazard ratio):ti,ab,kw (Word variations have been searched) | 327262 |
| #17 | (Disease Control Rate):ti,ab,kw OR (DCR):ti,ab,kw (Word variations have been searched) | 155965 |
| #18 | (HR):ti,ab,kw OR (overall survival):ti,ab,kw OR (progression-free survival):ti,ab,kw OR (Overall Response Rate):ti,ab,kw OR (ORR):ti,ab,kw (Word variations have been searched) | 153134 |
| #19 | #2 or #3 or #4 or #5 or #6 or #16 or #17 or #18 | 614907 |
| #20 | (depress*):ti,ab,kw OR (Anxiety):ti,ab,kw OR (chronic stress):ti,ab,kw OR (stress):ti,ab,kw OR (psychological distress):ti,ab,kw (Word variations have been searched) | 249742 |
| #21 | (emotional distress):ti,ab,kw OR (mental disorder):ti,ab,kw OR (depressive disorder):ti,ab,kw (Word variations have been searched) | 89080 |
| #22 | #7 or #8 or #9 or #10 or #11 or #20 or #21 | 329222 |
| #23 | #15 and #19 and #22 in Trials | 302 |

**Supplementary Table S4.** **Search strategy for Web of Science**

| #1 | Carcinoma, Non-Small-Cell Lung (Topic) OR Carcinoma, Non Small Cell Lung (Topic) OR Non-Small-Cell Lung Carcinomas (Topic) OR Carcinoma, Non-Small Cell Lung (Topic) OR Non-Small Cell Lung Cancer (Topic) OR Non-Small-Cell Lung Carcinoma (Topic) OR Non Small Cell Lung Carcinoma (Topic) OR Nonsmall Cell Lung Cancer (Topic) OR Non-Small Cell Lung Carcinoma (Topic) OR Non small Cell Lung Cancer (Topic) OR NSCLC (Topic) | 166,104 |
| --- | --- | --- |
| #2 | survival (Topic) OR mortal* (Topic) OR death (Topic) OR prognos* (Topic) OR hazard ratio (Topic) OR HR (Topic) OR overall survival (Topic) OR progression-free survival (Topic) OR Overall Response Rate (Topic) OR ORR (Topic) OR Disease Control Rate (Topic) OR DCR (Topic) | 5,499,166 |
| #3 | depress* (Topic) OR anxiety (Topic) OR chronic stress (Topic) OR stress (Topic) OR psychological distress (Topic) OR emotional distress (Topic) OR mental disorder (Topic) OR depressive disorder (Topic) | 4,407,026 |
| #4 | #1 AND #2 AND #3 | 1,662 |

**Supplementary Table S5. Search strategy for PsycINFO**

| 1 | (title: "Carcinoma, Non-Small-Cell Lung" OR title: "Carcinoma, Non Small Cell Lung" OR title: "Non-Small-Cell Lung Carcinomas" OR title: "Carcinoma, Non-Small Cell Lung" OR title: "Non-Small Cell Lung Cancer" OR title: "Non-Small-Cell Lung Carcinoma" OR title: "Non Small Cell Lung Carcinoma" OR title: "Nonsmall Cell Lung Cancer" OR title: "Non-Small Cell Lung Carcinoma" OR title: "Non small Cell Lung Cancer" OR title: "NSCLC" OR abstract: "Carcinoma, Non-Small-Cell Lung" OR abstract: "Carcinoma, Non Small Cell Lung" OR abstract: "Non-Small-Cell Lung Carcinomas" OR abstract: "Carcinoma, Non-Small Cell Lung" OR abstract: "Non-Small Cell Lung Cancer" OR abstract: "Non-Small-Cell Lung Carcinoma" OR abstract: "Non Small Cell Lung Carcinoma" OR abstract: "Nonsmall Cell Lung Cancer" OR abstract: "Non-Small Cell Lung Carcinoma" OR abstract: "Non small Cell Lung Cancer" OR abstract: "NSCLC") AND (title: "Survival" OR title: "Mortality" OR title: "Prognosis" OR title: "Survival Analysis" OR title: "Risk Assessment" OR title: survival OR title: mortal* OR title: death OR title: prognos* OR title: "hazard ratio" OR title: "HR" OR title: "overall survival" OR title: "progression-free survival" OR title: "Overall Response Rate" OR title: "ORR" OR title: "Disease Control Rate" OR title: "DCR" OR abstract: "Survival" OR abstract: "Mortality" OR abstract: "Prognosis" OR abstract: "Survival Analysis" OR abstract: "Risk Assessment" OR abstract: survival OR abstract: mortal* OR abstract: death OR abstract: prognos* OR abstract: "hazard ratio" OR abstract: "HR" OR abstract: "overall survival" OR abstract: "progression-free survival" OR abstract: "Overall Response Rate" OR abstract: "ORR" OR abstract: "Disease Control Rate" OR abstract: "DCR") **AND (title: "Depression" OR title: "Anxiety" OR title: "Stress, Psychological" OR title: "Mental Disorders" OR title: depress* OR title: anxiety OR title: "chronic stress" OR title: stress OR title: "psychological distress" OR title: "emotional distress" OR title: "mental disorder" OR title: "depressive disorder" OR abstract: "Depression" OR abstract: "Anxiety" OR abstract: "Stress, Psychological" OR abstract: "Mental Disorders" OR abstract: depress* OR abstract: anxiety OR abstract: "chronic stress" OR abstract: stress OR abstract: "psychological distress" OR abstract: "emotional distress" OR abstract: "mental disorder" OR abstract: "depressive disorder"**) | 30 |
| --- | --- | --- |

**Supplementary Table S6. Quality assessment of cohort studies included according to the Newcastle–Ottawa Scale**

| Study | Selection | Comparability | Outcome |
| --- | --- | --- | --- |
| Tatsuo Akechi et al. (2001) | **** | / | * |
| William F. Pirl et al. (2008) | *** | * | *** |
| Mei-Ling Chen et al. (2011) | **** | ** | *** |
| Oscar Arrieta et al. (2013) | **** | ** | ** |
| Jue Chen et al. (2015) | *** | / | *** |
| Zi-Ran Bi et al. (2022) | *** | * | ** |
| Yue Zeng et al. (2024) | *** | ** | *** |
| *, indicates that the study met the criterion; /, not stated; | | | |

**Supplementary Table S7. Quality assessment of randomized controlled trials included according to the Cochrane Risk of Bias 2.0 tool**

| **Study: William F. Pirl et al. (2012)** | |
| --- | --- |
| **Domain 1:**Bias arising from the randomization process | **Some concerns** |
| **Domain 2:** Bias due to deviations from intended interventions | **Low risk** |
| **Domain 3:** Bias due to missing outcome data | **Low risk** |
| **Domain 4:** Bias in measurement of the outcome | **Some concerns** |
| **Domain 5:** Bias in selection of the reported result | **Low risk** |
| **Overall risk of bias** | **Some concerns** |

**Supplementary Table S8. Evidence profile for emotional distress and clinical outcomes in advanced non-small cell lung cancer**

| **№ of studies** | **Certainty assessment** | | | | | | **Effect** | | | **Certainty** | **Importance** |
| --- | --- | --- | --- | --- | --- | --- | --- | --- | --- | --- | --- |
|  | **Study design** | **Risk of bias** | **Inconsistency** | **Indirectness** | **Imprecision** | **Other considerations** | **№ of events** | **№ of individuals** | **Relative (95% CI)** |  |  |
| Overall Survival (follow-up: range 6 months to 26.8 months; assessed with: HR) | | | | | | | | | | | |
| 6 | cohort studies | not serious | not serious | not serious | not serious | none | / | 911 | HR 1.85  (1.50, 2.27) | ⊕⊕OO Low | CRITICAL |
| Progression-Free Survival (follow-up: range 12 months to 16 months; assessed with: HR) | | | | | | | | | | | |
| 3 | cohort studies | serious^1^ | serious^2^ | not serious | not serious | none | / | 457 | HR 1.80  (1.22, 2.66) | OOOO  Very low | CRITICAL |
| Objective Response Rate (follow-up: range 6 months to 16 months; assessed with: OR) | | | | | | | | | | | |
| 3 | cohort studies | not serious^1^ | not serious | not serious | not serious | none | 229 | 546 | OR 0.55 (0.37, 0.80) | ⊕⊕OO Low | CRITICAL |
| CI, confidence interval; HR, Hazard Ratio; OR, Odds Ratio | | | | | | | | | | | |

#### Explanations

1 Due to inadequate adjustment for critical confounding factors.

2 I^2^= 63%.
