# Supplementary material for: Prognostic value of emotional distress in advanced non–small cell lung cancer: a systematic review and meta-analysis
Source: Front Oncol. 2026 May 20;16:1836242. doi: 10.3389/fonc.2026.1836242 (PMC13230033; doi:10.3389/fonc.2026.1836242)
Supplement: Supplementary Figure 1 — Quality assessment of studies included according to the Newcastle–Ottawa Scale. [file DataSheet1.docx]

**A systematic review and meta-analysis on the prognostic value of emotional distress in non-small cell lung cancer patients**

| **Legend** |  | **Pages** |
| --- | --- | --- |
| Supplementary Figure S1 | Quality assessment of studies included according to the Newcastle–Ottawa Scale | 2 |
| Supplementary Figure S2 | Quality assessment of randomized controlled trials included according to the Cochrane Risk of Bias 2.0 tool | 3 |
| Supplementary Figure S3 | Effect of emotional distress on overall survival by timing of ED assessment | 4 |
| Supplementary Figure S4 | Effect of emotional distress on overall survival by age subgroup | 5 |
| Supplementary Figure S5 | Sensitivity analysis for overall survival | 6 |

**
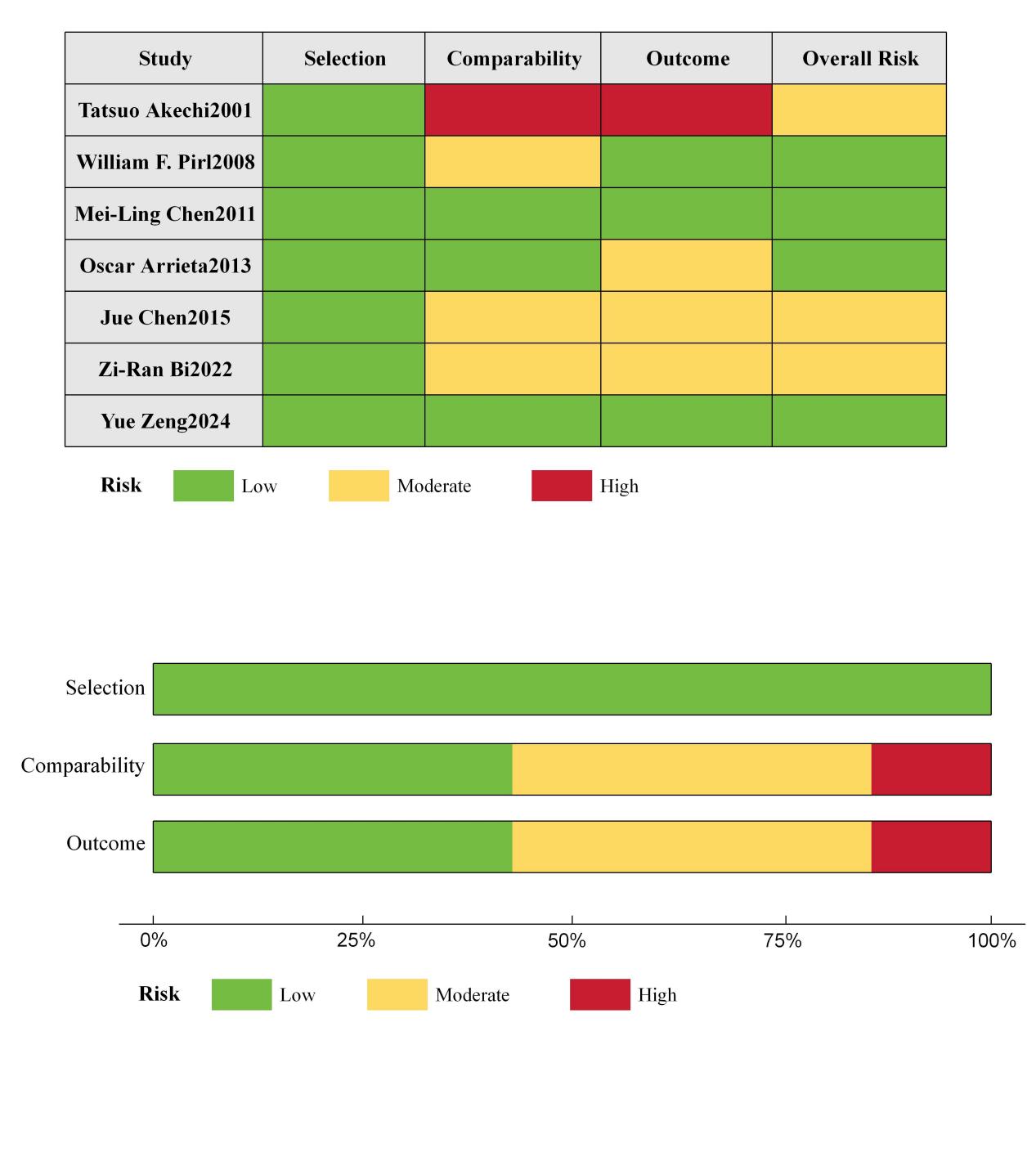
**

**Supplementary Figure S1. Quality assessment of studies included according to the Newcastle–Ottawa Scale**

**
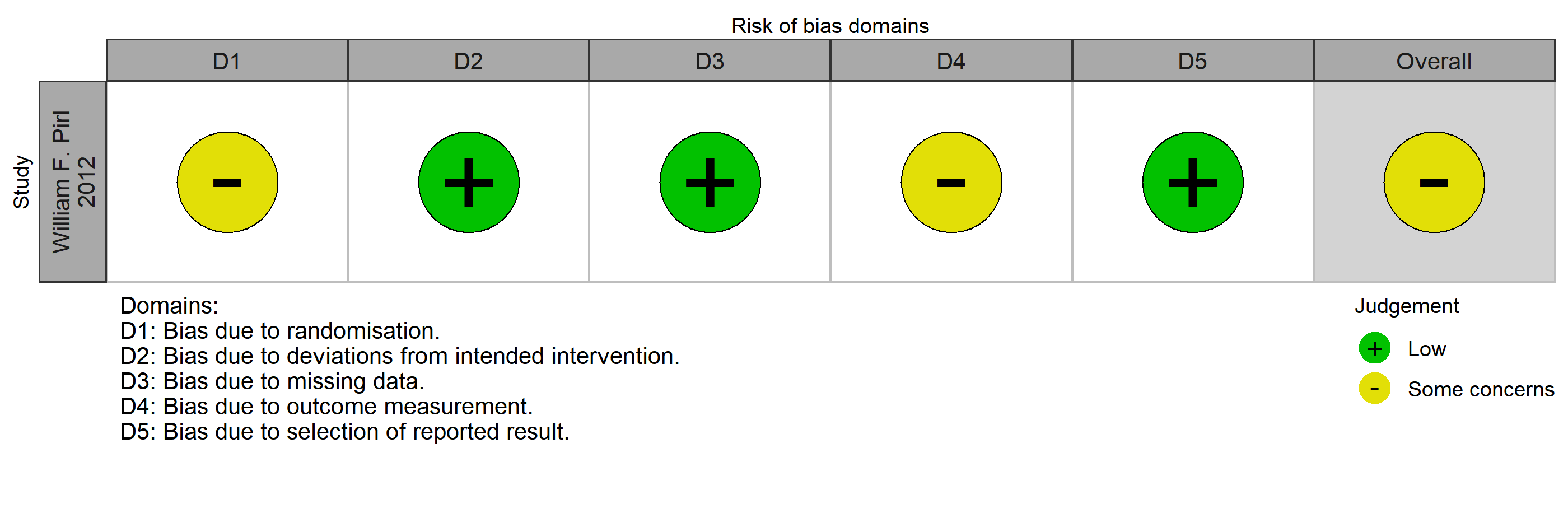
**

**Supplementary Figure S2. Quality assessment of randomized controlled trials included according to the Cochrane Risk of Bias 2.0 tool**

**
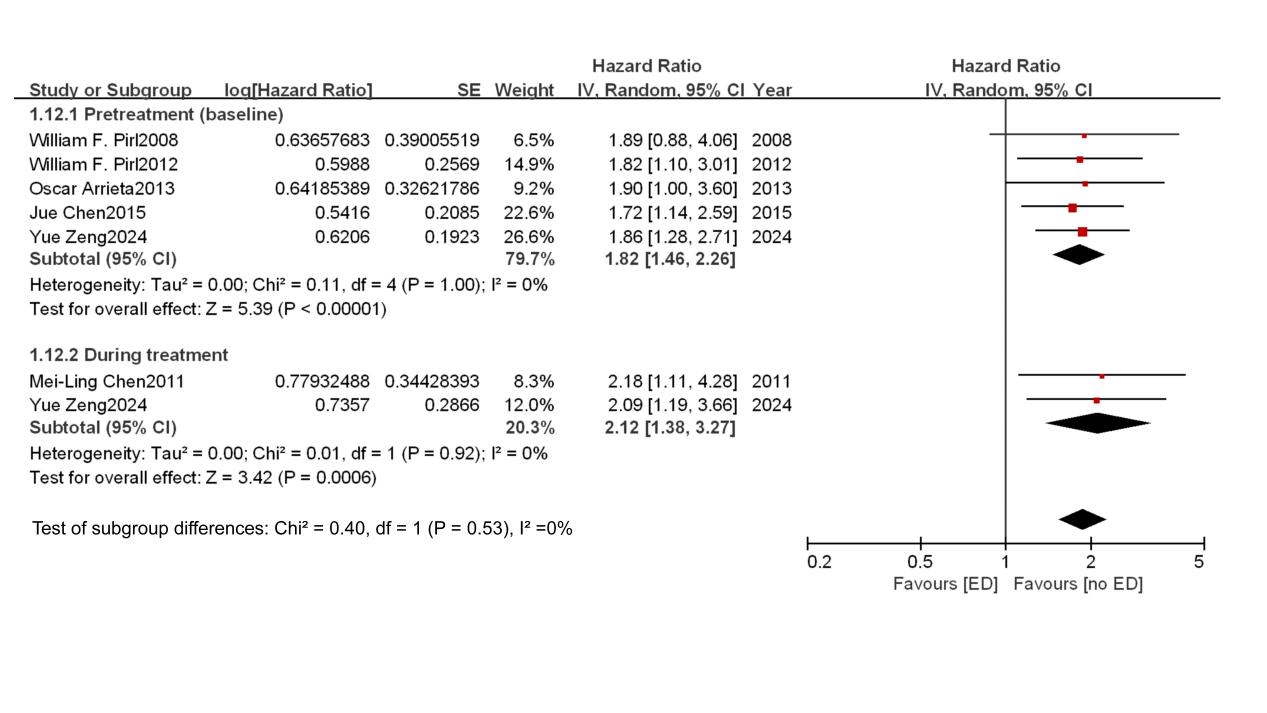
**

**Supplementary Figure S3. Effect of emotional distress on overall survival by timing of ED assessment**

**Supplementary Figure S4. Effect of emotional distress on overall survival by age subgroup**


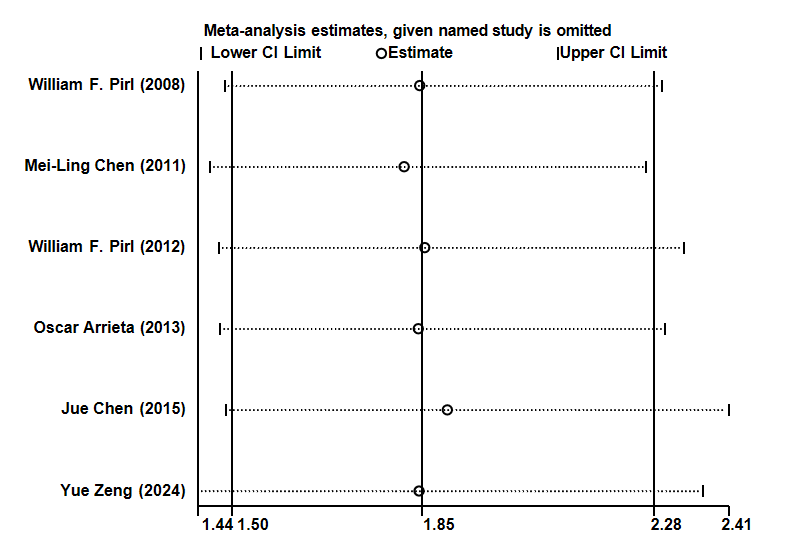


**Supplementary Figure S6. Sensitivity analysis for overall survival, performed by iteratively removing one study to assess the robustness of the pooled hazard ratio**
